# Are Special Care Dentistry Services Prepared for a Global Disruption in Healthcare? A Call for a Wider Promotion of Dental Conscious Sedation Training

**DOI:** 10.3390/healthcare8040419

**Published:** 2020-10-22

**Authors:** Arkadiusz Dziedzic, Marta Tanasiewicz, Hassan Abed, Chris Dickinson, Bruna Picciani

**Affiliations:** 1 Department of Conservative Dentistry with Endodontics, Medical University of Silesia, 40-055 Katowice, Poland; martatanasiewicz@sum.edu.pl; 2 Department of Sedation and Special Care Dentistry, King’s College, Guy’s Hospital, London SE1 9RT, UK; hassan.abed@kcl.ac.uk (H.A.); chris.dickinson@kcl.ac.uk (C.D.); 3 Graduate Program in Dentistry, Federal Fluminense University, Nova Friburgo, Dental Center for Patients with Special Needs, Rio de Janeiro 24220-008, Brazil; brunapicciani@yahoo.com.br

**Keywords:** COVID-19, conscious sedation, community dental care, special dental care, education, continuous professional development, anxiety

## Abstract

Recently, calls for prompt and smart reform of dental education and postgraduate training have been made, reflecting the current global healthcare needs and addressing the most common problems faced by dental care providers. Objectives: Herewith, we propose the enhancement of multilevel dental training in dental conscious sedation (DCS), in order to meet the increasing demands associated with current and post-pandemic times. The temporary suspension of general anaesthesia and hospital-based sedation provision in response to coronavirus disease 2019 (COVID-19) revealed the urgent need for more efficient utilization of a variety of forms of DCS. Whilst the global spread of Severe Acute Respiratory Syndrome novel coronavirus (SARS-CoV-2) has particularly challenged dental sedation teams in community services, the appropriate preparation for similar disruptions in future should be undertaken proactively. In response, dental schools and commissioners are obliged to implement innovations in teaching, with the development of new programs supporting trainer–trainee interactions and focusing on practical sedation skills. Conclusions: The joint efforts of educators, healthcare providers, and commissioners, as well as adequate and robust DCS training utilizing a variety of teaching methods, would allow our profession to face the growing demand for pain and anxiety control measures in light of the current situation, which may increase even further over time. Decision makers are urged to consider making training in DCS more accessible, meeting current healthcare demands, and equally providing essential support for the special dental care sector.

## 1. Introduction

Uninterrupted and efficient special care dentistry (SCD) provision depends upon the competent dental team members, their joined, mixed practical skills and independent arrangement, along with close cooperation with other specialists and medical professionals. The worldwide spread of the coronavirus disease 2019 (COVID-19) pandemic announced in early 2020 and the globally implemented lockdown measures that followed, highlighted the various limitations of dental services, in which the range of treatment provided in hospital setting had to be dramatically reduced. What is more, dental schools around the world were forced to defer academic dental education programs and specialist clinical training in all dental disciplines, including special care dentistry, which requires on-site clinical sessions in delivering sedation techniques under direct clinical supervision [1,2]. The temporary suspension and/or global reduction of general anaesthesia (GA) sessions and specialty training in anaesthesia, as well as deferred hospital-based sedation provision, along with a shortage of sedation-trained hospital personnel and limited alternatives of anxiety management options for dental patients revealed the urgent need to be better prepared for mass healthcare disruption and any such situation in future [3]. 

Whilst the uninterrupted provision of adequate anxiety control is an integral element of clinical dentistry, the utilization of a variety of forms of dental conscious sedation (DCS) is required, which comprises oral, intranasal/transmucosal inhalation, and intravenous sedation, or hybrid and combined methods [4,5,6] (Table 1). The therapeutic goals of sedation in the dental and medical field are anxiety relief, reduction of psychological stress, and post-operative amnesia for traumatic procedure or event. In DCS, verbal contact with the patient and physiological reflexes are maintained, in contrary to deep sedation and general anaesthesia. This should also involve the wider implementation of pre-procedural anxiolytic medications (benzodiazepine pre-medication), if needed for less complex cases. Moreover, clinicians should also consider the use of non-pharmacological methods of behavioural management techniques selected individually as an alternative and/or adjunct to DCS. It must be emphasized that a “one method fits all” approach is deemed inappropriate, as this rule is particularly valid for DCS. 

In the past, major disruptions in the healthcare system induced a rapid development of innovative ways and measures of patients’ management to overcome the sudden health, and organizational burden to the staff. It is not surprising that, similarly, the SARS-CoV-2 global spread has particularly challenged dental sedation teams, community services, and those in hospital care, evoking various response methods worldwide [7]. In the field of dental and medical education, all aspects of training programs have been affected, with impaired learning of supervised procedures, suspended educational activities, and altered clinical sessions [3]. That is why appropriate preparation for similar problems and disruptions in the future should be proactively undertaken. It is our duty to make a professional commitment to respond quickly to challenges that will arise, so we can meet our patients’ needs in the most effective way.

Despite of the fact that the COVID-19 outbreak has caused a mass-scale detrimental effect on healthcare provision [8], it might also bring the opportunity and impetus for dental sedation units/teachers in primary and secondary care, as well as in university departments, to improve or diversify their approaches. The aim would be to provide a variety of effective clinical and educational programs delivering outcomes appropriately applicable to updated undergraduate and postgraduate training in DCS. The current healthcare crisis has revealed an underestimation of the crucial role of and need for sedation in clinical dentistry. By learning this lesson and looking into the future, dental care providers and managers should now turn their attention to the long-term impact of health service disruption, predicting and preventing far more profound effects in case of any other similar epidemic outbreaks. As, according to health experts’ evaluations, DCS is the most cost-effective form of dental anxiety management, it needs to be better promoted and acknowledged among medical and dental professionals, with additional funding and options of a long-term career [9]. At the dental education level, a wider implementation of new concepts and tools of DCS teaching on a broader scale would be much welcome, regardless of the current, sudden impact of the global pandemic. In general, the enhanced teaching of DCS could be introduced in any healthcare institution, at various levels of undergraduate and postgraduate dental training. The broader use of a sedation-focused model of special care dentistry (SCD) is easily applicable within both primary dental care and specialist secondary services. 

## 2. Professional Guidance and Recommendations for the Use of Dental Sedation during COVID-19 Pandemic

There is still some confusion within the dental profession regarding the use of certain DCS techniques during the current epidemic/pandemic crisis. This uncertainty will inevitably produce restriction of use and, thus, disadvantage patients’ care as clinicians may remain on the side of avoidance until the situation is clarified. Since March 2020, changes have been implemented in the provision of pain and anxiety management due to organizational adjustments and cross-infection control requirements. This situation highlighted the crucial role of up-to-date guidance and involvement of professional authorities in ensuring the safety of DCS use in all patients but is especially important for vulnerable groups of patients, often medically compromised, at the time of the current pandemic. Moreover, it has resulted in an increased need for attention to be paid to the safe provision of conscious sedation instead of reliance on general anaesthesia. Different advisory and regulatory professional bodies and professional societies made bold attempts to provide regular updates concerning the use of DCS for dental professionals, to ensure a safe provision of affected dental care [10]. However, this advice and guidance did not always send a consistent message and, what is more, it placed dental sedation providers and educators in an invidious position, affecting dentally anxious patients who required urgent care. 

The updated guidance for DCS, similarly to the existing one [11], is expected to be applicable to all dental providers, i.e., dental practice, public or community dental service, or hospital setting, in order to facilitate the provision of any type of dental treatment, especially invasive ones (extraction). Essentially, it aims to be directed at all dental and healthcare professionals, including anaesthetists and medical sedationists, involved in the care of patients receiving conscious sedation for dental care in any setting. This DCS will also be of relevance to those involved in dental education, undergraduate training, and commissioners of services. In the United Kingdom, the recently updated guidance for dental care providers released by the Public Health of England, Society for the Advancement of Anaesthesia in Dentistry (SAAD) and issued by the Royal College of England encourages safe clinical practice through the provision of recommendations and practical advice. For some aspects of the provision of conscious sedation for dentistry during the pandemic crisis, little evidence to inform the recommendations is found. The trustees of SAAD work with the U.K. government, academic professionals, regulatory bodies, as well as public health authorities. SAAD has clearly reiterated the position of the Chief Dental Officer concerning the safety of using inhalation sedation during COVID-19 pandemic, as it is not considered an aerosol-based procedure. From the infection control point of view, conscious sedation in the forms of inhalation and intravenous (IV) single-drug sedation does not appear to carry any higher procedural risk of microbiological contamination, or droplet-born infection than any form of dental treatment. The recent SAAD and Chief Dental Officer, U.K., statements are clear that “sedation is an excellent adjunct as you are restarting your practice. You should consider using it because it is safe to do so” [11] and, “inhalation sedation is not considered an aerosol generating procedure (AGP) and may be a suitable alternative to general anaesthesia” [12]. Further guidance on the provision of safe intravenous sedation during the current pandemic has been posted on their website. 

**Figure 1:**
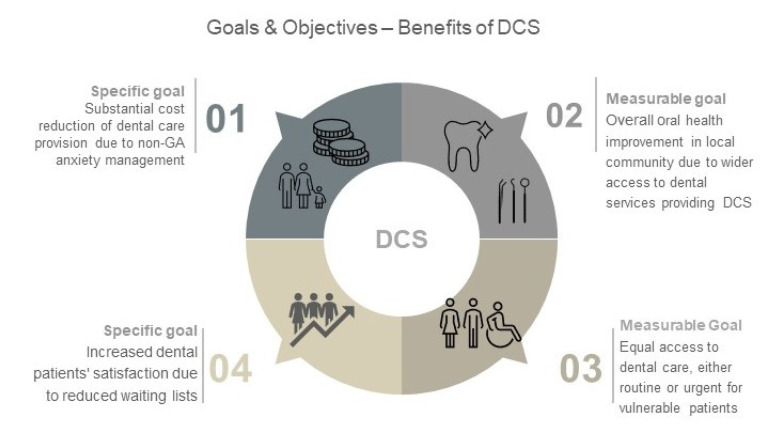
The benefits of dental conscious sedation in primary healthcare setting.

This clearly emphasizes that DCS should be considered an essential element of elective and urgent dental care during any epidemic/pandemic time. Equally, appropriate DCS techniques should be individually selected for various categories of special care patients, including people with learning disabilities, co-morbidities, and dental anxiety. Vulnerable patients have significant barriers to accessing oral healthcare and difficulties in obtaining DCS should not be an additional barrier; hence, this is especially pertinent where DCS techniques can be a viable alternative to general anaesthesia. That is why we must make as much effort as possible to minimize the restrictive consequences of the current situation for patients with special needs, who often rely on some form of sedation to enable and secure their dental health (Figure 1).

## 3. Transition from a General Anaesthesia-Based Model to the Use of Various Dental Sedation Methods for Anxiety Management

The transition from a general anaesthesia-based model to the use of various dental sedation methods for anxiety management had been gradually evolving over the last decades as a result of improved healthcare protocols. The SARS-CoV-2 outbreak inevitably intensified and accelerated these general trends in dental care provision, which were already commenced and recommended. Considering universal equality legislation across the world, the widely available access to DCS should be considered an obligation imposed on dental provider rather than an “adjunct and exceptional measure”. All patients, regardless of their origin, nationality, and medical status, deserve appropriate anxiety management measures, especially for invasive dental procedures. Unfortunately, this approach may not always be applicable as training in SCD and DCS vary considerably in different countries, also there is no international consensus for DCS teaching or clinical provision. What is more, these differences in dental training are in fact often associated with the “historical context”’ of dental education and/or the influence of professional bodies, with a substantial impact of justified opinions provided by experts in anaesthesiology. However, we are convinced that collective global efforts should be put in place to support appropriate diversified training, audit, and evaluation of DCS, which should be considered an impactful adjunct method in dentistry, working alongside and also supporting dental GA service provision when required. For some groups of SCD patients, dental treatment under GA remains a viable option for the provision of efficient healthcare, and in selected cases, dental general anaesthesia (DGA) can often be the only alternative for the persons who are unable to cope with routine dental treatment provided by any other means [13].

**Figure 2:**
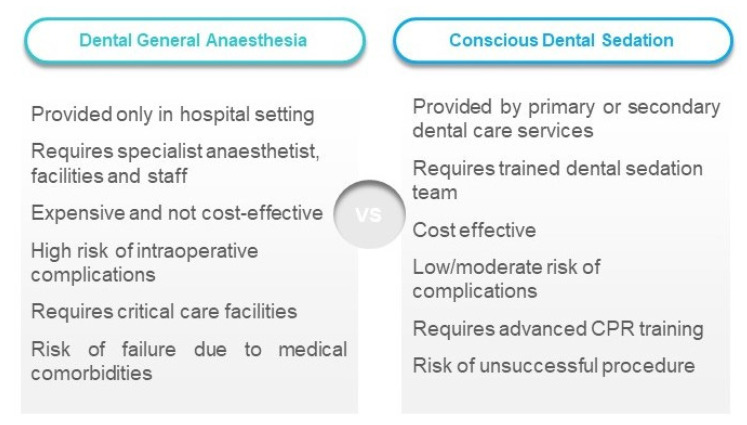
Comparison of general anaesthesia provision in dental care vs. dental conscious sedation.

It is challenging for clinicians to cope with cases of non-cooperative patients with special requirements and phobic individuals when advanced options for anxiety management are considerably reduced. Although dental treatment under GA in hospital setting seems to have some essential benefits, such as appropriate critical care and recovery facilities, highly trained staff, controlled and monitored conditions during a single treatment session, DGA should be only considered as a “last resort option”. Whilst general anaesthesia services during the pandemic crisis period were deferred or severely restricted to the most urgent/emergency cases, the use of DCS can be extremely valuable in taking pressure off from limited GA service provision for urgent dental care (Figure 2). Without well-organized and managed, DCS service provision, supported financially and logistically by authorities, ministries of health, and local healthcare decision-makers, GA provision becomes the only (limited) alternative in a particular region or even in the whole country. This could undoubtedly severely affect the access to dental care, anxiety control measures, and additional costs of healthcare. The overall cost of treatment under GA is 3–4 times higher than that of utilizing DCS methods and includes remuneration for anaesthetists, dental staff, as well as the cost of consumables, equipment, portering, and the availability of inpatient beds reserved for use by the service [14]. The retrospective study assessing the need for DGA with Oldham Community Dental Service demonstrated that only 25% of patients actually required a single session of DGA, and the rest of the group accepted dental treatment with routine means, local anaesthesia, and/or conscious sedation [15]. Unfortunately, there are no randomized controlled studies comparing general anaesthesia versus sedation for children and adolescents undergoing dental treatment. 

It should be noted that dental general anaesthesia continues to have a well-established place in the management of special dental care as a “last resort” option, although, its role primary role should be limited to selected and most challenging cases in interdisciplinary dental care provision. GA will never be fully substituted by even the most advanced dental conscious sedation techniques, although its use is going to be reduced for treating dental patients. It is worth mentioning that recently anaesthetists and other medical professionals urgently called for a wider use of regional anaesthesia instead of the “traditional” way of procedural anaesthesia during pandemic time [16].

In the United Kingdom, the Department of Health (England) report published in 2000 ordered the confinement of GA to a hospital setting that has critical care facilities, with the immediate effect that GA cannot be provided within the primary care setting and must only be applied by a registered specialist anaesthetist, a trainee working under supervision as part of the Royal College of Anaesthetists, or a non-consultant career-grade anaesthetist under the supervision of a named consultant anaesthetist [17,18]. Inevitably, there is the significant issue of cost, where DCS provision is much more cost-effective compared to GA service provision restricted to hospital-based services (Figure 2). DCS can be provided independently by local sedation teams in the primary/secondary care setting and this would support people who require anxiolytic measures, those who might benefit from sedation for medical reasons, or for vulnerable people with learning difficulties and disabilities. Furthermore, it is important that a wide margin of safety in terms of side effects is maintained during DCS, compared to that needed in the unconscious state in case of deep or general anaesthesia. Adequate and appropriate conscious sedation techniques should prevent the necessity of rescheduling procedures when sedation is unsuccessful. What is more, taking into consideration the valid, informed consent aspect required by law, an anxious child who might benefit from dental care under sedation under the age of 16, and who is deemed to be “Gillick competent”, i.e., is considered mature enough to be able to satisfy the requirements of informed consent, can provide an individual consent for treatment under sedation without the requirement for parental consent. Although it is always recommended to involve the parents/legal guardian in the consent process for a Gillick competent child under the age of 16 [17].

The sedation-based concept of special dental care relies on the modern approach, which restricts the use of GA to the minimum and in which the provision of care is concentrated on the patient’s specific needs, with careful selection of dedicated sedation methods. This new approach evolved over the last decades to allow a gradual shift from hospital-centred dental care provision to a highly individual way of managing patients, supporting them by utilizing various techniques for anxiety management, at both primary and secondary levels. This transition from a general anaesthesia-based model of paediatric and special care dentistry provision to the use of various sedation methods (inhalation, intravenous, oral, intranasal, or combined/hybrid techniques) in outpatient dental clinics should be implemented more widely, regardless of the COVID-19-induced pandemic crisis, addressing the challenge of pain and anxiety management in most severe cases [19]. Nevertheless, the COVID-19 healthcare crisis has certainly accelerated the need for wider use of DCS for dental patients, rapidly highlighting the necessity of global changes in dental education. Whilst increasing demand for pain- and anxiety-free dental care as well as the increasing prevalence of medial co-morbidities in ageing populations, among decision-makers, in academic circles, dental schools, and training centres/independent providers should be encouraged to consider implementation of the proposed changes at a variety of levels.

## 4. Enhancement of Dental Conscious Sedation Students’ Curricula

Universities, dental schools, and university hospitals play an essential role in DCS teaching and structured practical training and, therefore, in its ultimate provision outside of the teaching institutions. Nowadays, dental educators and their commissions for change should begin advocating for dental curricula reform, in order to develop in students the adjustable skills required for the contemporary practice of dentistry, including DCS. In some countries, including the United Kingdom, standard methods with the use of either inhalation sedation (nitrous oxide/oxygen) or intravenous midazolam alone, are taught at undergraduate dental school level and are suitable for dentists to perform in primary care provided they work within their competencies and keep their knowledge, experience, and training in such techniques regularly updated. Hence, in light of the current pandemic, the DCS and SCD curricula and supervised clinical practice at undergraduate and postgraduate levels should be enhanced and increased worldwide, with primary support of DCS teaching units. Additional clinical sessions and hands-on DCS training at various dental departments, with a crucial role of disciplines such as oral surgery, paediatrics, and special care dentistry might encourage students to develop a deeper interest in DCS and be more involved after graduation. Moreover, advanced training in DCS could be more widely promoted as part of an interdisciplinary curriculum in a variety of specialist dental subjects, such as restorative, implant, and other surgical and non-surgical disciplines, including integrated and special care dentistry, which have already been introduced. The use of modern methods and equipment for preclinical, and clinical training in DCS, such as virtual reality, simulators, and case reflection would facilitate the acquisition of essential practical skills, including basic skill intravenous cannulation [20,21]. Examples of educational methods that can be utilized during under- and postgraduate DCS training are presented in Table 2. To be appropriate, this comprehensive educational approach in dental study must be considered for each country having specific regulations and legislative measures. National education and training bodies, as well as regulators should provide flexibility in their requirements. In general, the ultimate learning objectives during DCS training have to comprise, among others, safe patient preparation and adequate clinical assessment, safe preparation of the environment/choice of equipment, understanding of levels of monitoring for safe sedation, safe practice in drug administration, recognition and management of complications of sedation, and demonstration of assessment for safe patient discharge [22]. The detailed aims should involve the following: inclusion/exclusion clinical criteria, risk assessment, assessment of individual need for sedation, informed consent, pharmacological characteristics of sedative drugs, their advantages and disadvantages, management of medical emergencies, intraoperative monitoring, and practical aspects (e.g., venous access). Taking into consideration a student/trainee’s personal profile, apart from the main “technical requirements and assets”, leadership team’s management skills are equally important, especially when a medical emergency or crisis situation occurs. 

Apparently, the time is right for the academic community to pursue a serious discussion about DCS evolution, more training places, and nationally funded curricula, also during online deanery briefings at national academic level. A proposal for change dedicated to the faculty authorities might be considered by students’ representatives, along with requesting better promotion of DCS in the course of undergraduate training. By presenting rational and factual arguments, a real opportunity is provided to enhance the DCS teaching environment at the academic level, which would be beneficial for further primary, secondary, and tertiary dental care.

## 5. Broader Access to Postgraduate Dental Training for Newly Graduated Dentists and Experienced Practitioners. Promotion of Local On-Site Training in DCS 

**Figure 3:**
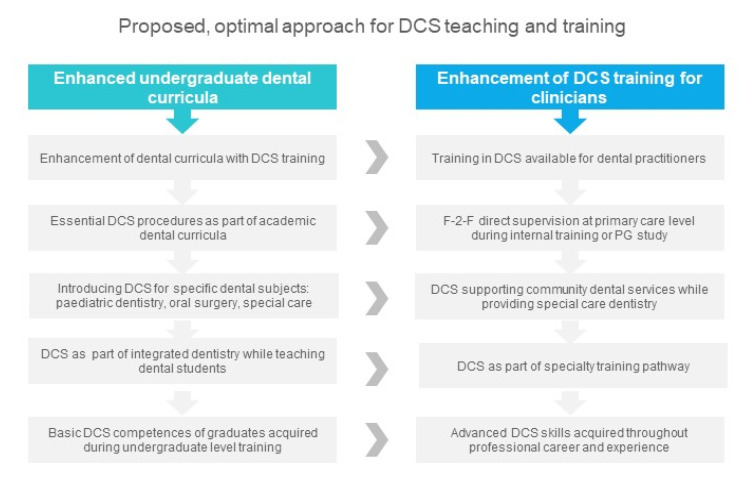
Proposed optimized approach for enhanced dental conscious sedation teaching and training.

Dental professionals are always eager to master new skills and gain knowledge relevant for their workplace; unfortunately, the options for further training at university level can be quite limited. We would welcome and encourage dental departments, as well as national societies and organizations all over the world to support continuous professional development in lifelong learning mode, with structured DCS training for dental practitioners, in the form of postgraduate studies in DCS (certificate/diploma). In the U.K., professional bodies such as SAAD, the British Society of Disability and Oral Health (BSDH), and the Royal Colleges have a well-established history of continuous support for dental practitioners. Moreover, specialty-orientated postgraduate curricula in dental subjects might be extended and enhanced with the expertise in practical application of DCS for diverse groups of patients. The changes we have seen in society enforced upon populations by the COVID-19 pandemic have shown the innovations that can happen through adversity. The rise in online communication during this time demonstrated that the theoretical aspects of many training programs may be delivered through distance learning and hybrid/blended learning formats. What is more, implementation of novel, combined, and/or advanced DCS techniques [23,24] by primary/secondary dental care providers is likely to diminish the impact of limited GA sessions and the overall burden of restricted access to the hospital setting as a result of any epidemic scenario in future, if it occurs. The new DCS postgraduate training curriculum model ought to comprise specific and unique elements managed by interdisciplinary consultants and faculty experts. The process of DCS training reform aims to move from a traditional isolated curriculum to one that uses integrated multidisciplinary courses to improve teaching outcome and practical skill acquisition, being beneficial for community and special dental care teams (Figure 3). This reformed curriculum should incorporate inter- and multidisciplinary courses, case-based active learning strategies, and concepts from continuous professional development theory [25]. Perhaps, a disruptive innovation in education might be required to alter the well-established model to improve the existing standards in DCS training. In fact, the provision of DCS skill sets is best achieved by on-site training, delivered locally, arranged by local dental teams, groups of enthusiastic professionals, and senior colleagues who can share their experience and knowledge. These practical skill sets can often only be taught with face-to-face (F-2-F) methodology, but the use of blended learning may reduce the difficulties of attending courses by limiting the amount of F-2-F teaching required. By raising the importance profile of DCS, organizations may be urged to support and train staff members in order to enhance their clinical skills and provision of DCS to the targeted groups of vulnerable patients. This is especially important and applicable in community dental service, where sedative techniques can be extremely useful, it may also encourage senior staff to become mentors to staff new to DCS techniques.

## 6. Dedicated Support for Research with Direct Relevance to Dental Conscious Sedation

The COVID-19 pandemic has had a major impact upon the research and study of DCS techniques, which will have been as affected as any other area of research. However, investigations into viable, safe, easily accessible sedative agents is vital at any time, especially during such unprecedented times as a pandemic, when the restriction of provision may adversely affect patient care. Furthermore, clinicians are willing to pursue their interest also towards research-oriented areas of expertise, introducing new concepts or clinical protocols, with the aim to improve the safety and efficiency of DCS. 

We would welcome innovative, integrated research assessing combined DCS techniques in the management of various groups of dental patients. Although a single-drug (midazolam) intravenous sedation has been established as the gold standard in a dental care setting due to its wide safety margin, studies in newer sedatives, such as dexmedetomidine and remimazolam [26,27] may produce distinct advantages over the traditional sedatives of midazolam and propofol, helping to resolve problems faced by dental services and related to the restrictions imposed due to the pandemic. It is expected that these modern sedative agents will be allowed to be used by anaesthetists as according to Consensus Statement of 21 European National Societies of Anaesthesia, due to well-known risks, even though propofol should be administered for procedural sedation only by those trained in general anaesthesia [28].

Without adequate focused research, we may never know the advantages or disadvantages that these drugs provide, which can be potentially utilized in an outpatient setting. The encouragement from national institutions responsible for research grants allocation seems to be a key aspect, with the outcome in the form of novel, better DCS methods that overcome the drawbacks of current DCS techniques. Nevertheless, comprehensive studies on sedative drugs and their interactions would provide a rational basis for more advanced sedation techniques. In addition, carefully designed and well-run randomized controlled studies are required to verify the effectiveness and safety of new DCS techniques versus GA in provision of dental treatment, particularly in children and adolescents.

## 7. Summary

**Figure 4:**
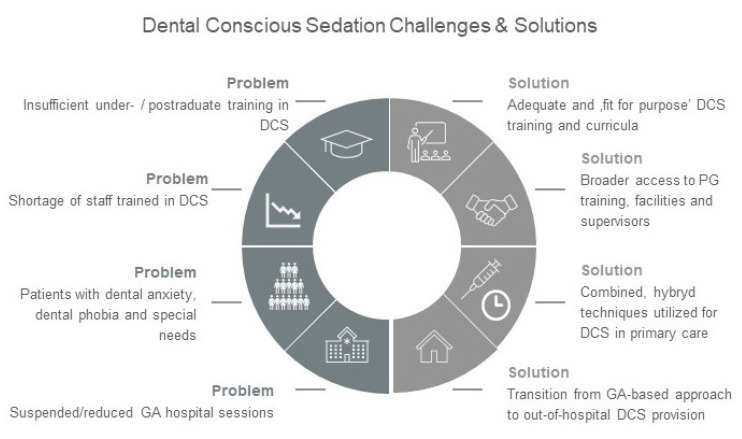
Provision of dental conscious sedation in primary and secondary care. Challenges and proposed solutions.

The COVID-19 pandemic has resulted in the biggest challenge that community and special care dental services have ever faced, and it has brought to light the shortfalls of DCS. This major disruption of healthcare should serve as a red flag that signals the need to enhance the way we manage patients with special needs. Challenges in the development and implementation of the reformed DCS provision, along with practical solutions, can be efficiently managed with the support of educators and healthcare commissioners supported with agenda for constructive changes (Figure 4). The more prepared dental sedation services are now, the more likely it is that any new crisis could have less impact on workflow and patient care; so, re-evaluation and re-adjustment is a key element of long-term success. Providing anxiety management during dental treatment is important for psychological and medical reasons, considerably reducing the risk of medical emergencies and supporting medically compromised patients [29]. Removing the possibility to provide DCS during the pandemic can adversely affect both. More importantly, as DCS is cost effective, easily learned, and well accepted by patients, it might be applicable across a wide variety of clinical situations and patient groups, regardless of their age and medical status.

The limited number of countries in which dental practitioners are allowed to provide basic IV sedation using a single sedative drug as operator–sedationist, without anaesthetist support, vastly hampers the access to DCS and severely disadvantages patients. Hence, this is the right time to initiate a global discussion with medical and dental representatives concerning dental training transformation and the wider use of DCS, particularly IV sedation, by dentists in different countries that currently restrict this feasible opportunity for various reasons. Predicting the possibility of other global healthcare disruptions, including epidemic outbreaks, the primary role of professional bodies and dental societies is to reaffirm the importance of future provision of comprehensive and “fit for purpose” dental services, which will be able to secure increasing healthcare demands in light of rapid demographic changes. The challenges of implementing wider access to DCS provision and consideration of subsequent DCS training developments aimed at facilitating their implementation should compose the practical solutions for the provision of DCS even during the most unpredictable time, such as a global pandemic. The results of changes in DCS provision should be considered to be directly applicable to any dental services across the world, with the caveat that sufficient administrative and educational efforts would be needed, and clinical demands would be defined for establishing primary-care-based sedation services locally, as an alternative to GA.

## 8. Conclusions

The broader provision of safe and effective dental conscious sedation requires adjustments in both further education of the profession and supportive clinical governance. By emphasizing the need for the wider implementation of dental sedation training programs for dental staff with special interest in anxiety management and special care dentistry, we can attract more attention from decision-makers. Adequate and robust dental conscious sedation training, utilizing a variety of flexible training methods would allow the profession to face the growing demand for pain and anxiety control measures in the light of current situation, which may potentially increase even further over time. We urge decision makers to consider making training in DCS for dental professionals more available and accessible in the future. We have become aware of the impact the COVID-19 crisis has had on sedation and general anaesthesia provision on global scale, and our duty is to make an adequate commitment to respond quickly to challenges that will arise, to meet our patients’ needs in the most effective way.

## Figures and Tables

**Table 1:** Comparison of the main advantages and disadvantages of various dental conscious sedation (DCS) techniques used in dental care services.

Conscious Sedation Method	Advantages	Disadvantages
Oral sedation (OS)	Simple method, does not require specific training/equipment, side effects are rare if any, can be combined with other DCS methods	Age restricted, unpredictable pharmacokinetics and patients’ response, limited efficiency of anxiolysis, risk of oversedation or undersedation, unpredictable sedative effect, sometimes prolonged recovery, may require a chaperone person
Inhalation sedation (IHS)	No age restrictions, although it requires good cooperation, wide safety margin, can be enhanced by additional techniques, such as music and tranquilizing verbal descriptions, quick recovery, does not require chaperone person	Limited efficiency, requires special, expensive equipment; anxiolysis less effective in adults; special facilities requirements (scavenging); operator sensitive; technique sensitive
Intravenous sedation with single-drug titration (midazolam IVS)	Effective in situations where OS or IHS failed or for patients with severe dental phobia who cannot be sedated with IHS, well-controlled patient’s response due to drug titration, allows a profound anxiolysis, post-operative amnesia effect, may substitute dental general anaesthesia in selected cases, reversal agent in case of intraoperative complications or abnormal reaction associated with the use of benzodiazepine agent	Age limited, usually not used in children below 12 y.o.a., risk of systemic complications, age-restricted, requires advanced training and cannulation skills, requires training in advanced life support, chaperone person required, comprehensive pre- and postoperative instructions for patient
Intranasal, transmucosal sedation with a short-acting benzodiazepine (midazolam)	Non-invasive, often used as an adjunct to IV sedation prior to cannulation, allowing a better patient’s co-operation during cannulation; does not require specific facilities or equipment; reversal agent in case of complications	Unpredictable sedative effect, unpleasant sensations during administration, usually not used alone, requires advanced training and cannulation skills (in case of adverse effects)

**Table 2:** Educational tools and various types of didactic methods used directly in DCS training.

Tool/Method	Educational Application and Features
Lecture: traditional, online, blended/mixed type	Mainly theoretical aspects, knowledge transfer with limited interaction
Student’s presentation: classroom, online	Direct involvement in a certain subject, deep understanding of required knowledge, communication skills development
Assignment: tentative, summative	Assessment of acquired knowledge and understanding
Case study reflection: classroom, online	Personal opinion about clinical case, with involvement of other students, learning from reflecting on past experience
Simulation: high-fidelity simulation with patient simulator and low-fidelity simulation with actors as simulated patients	Practical aspects of DCS without patient’s involvement
Workshop	Sharing experience, open discussion, exchange of thoughts and ideas, practical exercises
Virtual Reality Training	Expensive equipment, requires special software and knowledge/training, does not provide real interaction or tactile sensations, purely “technical feedback”
Clinical sessions	Practical training with patient’s presence, real interaction in clinical environment, communication skills practice
Peer Review and Research	Evidence-based aspects of DCS practice
Conference attendance	Variety of different topics, experts’ opinions, and experience, limited feedback
Self-learning (online)	Lack of interaction, no feedback, wide/uninterrupted access
